# Cyberchondria severity and quality of life among Lebanese adults: the mediating role of fear of COVID-19, depression, anxiety, stress and obsessive–compulsive behavior—a structural equation model approach

**DOI:** 10.1186/s40359-021-00674-8

**Published:** 2021-10-29

**Authors:** Clara Rahme, Marwan Akel, Sahar Obeid, Souheil Hallit

**Affiliations:** 1 Research Department, Psychiatric Hospital of the Cross, P.O. Box 60096, Jal Eddib, Lebanon; 2 School of Pharmacy, Lebanese International University, Beirut, Lebanon; 3 Faculty of Arts and Sciences, Holy Spirit University of Kaslik (USEK), Jounieh, Lebanon; 4 Faculty of Medicine and Medical Sciences, Holy Spirit University of Kaslik (USEK), Jounieh, Lebanon

**Keywords:** Cyberchondria, Quality of life, Anxiety, Stress, Obsession-compulsion

## Abstract

**Background:**

This study highlights the significant association between cyberchondria and quality of life among the Lebanese population in the time of COVID-19. The aim was to assess the association between cyberchondria and quality of life (QOL) of Lebanese community during the COVID-19 pandemic and assess the mediating effect of fear of COVID-19, depression, anxiety, stress and Yale-Brown Obsessive–Compulsive Scale in this association.

**Methods:**

This cross-sectional study was carried out between December 2020 and January 2021, during the COVID-19 pandemic. A total of 449 persons participated in this study by filling the online questionnaire. Structural equation modeling (SEM) was performed to examine the structural relationship between cyberchondria severity, the mediator (anxiety, stress, depression, obsessive–compulsive disorder (OCD) and fear of COVID-19) and physical/mental QOL.

**Results:**

Having a university level of education and older age were significantly associated with higher physical QOL scores, whereas higher obsession-compulsion disorder, higher stress and higher anxiety were significantly associated with lower physical QOL scores. Higher anxiety was significantly associated with lower mental QOL scores. The results of the SEM showed that stress, fear of COVID-19 and to a lesser limit OCD, mediated the association between cyberchondria severity and physical QOL, whereas anxiety, stress and fear of COVID-19 mediated the association between cyberchondria severity and mental QOL.

**Conclusion:**

This research reported interesting results encouraging more exploration of cyberchondria and its association with quality of life during this unique period of the pandemic. However, this virus has altered the lives of individuals all across the world, and the consequences will last for a long time. Along with all of the steps done to stop the development of COVID-19 and improve physical outcomes, mental health requires immediate care. More research is needed to determine the coping techniques people are employing to deal with the pandemic.

## Background

The current coronavirus pandemic is causing considerable psychological and physical stress worldwide [1]. Even though it has been easier than ever before to tackle the physical implications of infectious diseases, the psychological effects of such epidemics are pervasive and probably more severe. While fear has always been invoked by infectious diseases, and because of global access to information, this response has never been as widespread as it is with COVID-19 [2]. Therefore, at times of great instability, such as the COVID-19 pandemic, it is predicted that an increased number of cases of cyberchondria will occur [3]. By the appearance of the virus, media of all kinds were loaded with reports and analysis related to the causes and effects of the disease [4]. As a result, the internet has become an important global source of health information [2, 4] since it provides formal access to high quality, enormous volume, current and appropriate health information [5]. In the United States, for example, 35% of people use the Internet to search for medical problems that they or someone else may have [4]. Likewise, a study in 2016 found that more than 50% of adults in the United Kingdom were seeking online health information [4]. Considering the steady rise in Internet penetration across the world and the amount of information posted online, these trends are kind of expected. However, increased time spent searching for health information has amplified the levels of anxiety, a phenomenon called “Cyberchondria” [4, 6].

According to theoretical theories, a reinforcement cycle characterizes cyberchondria, in which an individual suffers increasing worry when accessing health information online, prompting them to seek reassurance regarding its accuracy. These attempts lead to a short-term drop in anxiety, which perpetuates the long-term cycle because anxiety reductions adversely promote information-seeking behaviors [7]. The reassurance-seeking model, which proposes that people with high levels of health anxiety participate in online health research to be comforted about their health concerns, was the first theoretical analysis of cyberchondria [8]. Because the structure of the Internet makes the outcome of reassurance seeking on the Internet entirely unpredictable, some people are reassured by what they find online, while others are not. Those who do not receive comfort or only receive partial reassurance and hence become more worried continue to use (online Health Research) OHR in an attempt to find reassurance [8]. The negative consequences of OHR, such as increased anxiety and discomfort, are linked to specific metacognitive beliefs. These beliefs could be about the Internet's ability to help people cope with health-related stress and worry (positive metacognitive beliefs), or they could be about the loss of control over OHR and a feeling that it is harmful (negative metacognitive beliefs) [8]. If negative metacognitive beliefs are more prevalent, OHR becomes a perceived threat, and OHR is viewed as stressful, obsessive, and out of control—a pattern known as “compulsive OHR” [8].

Cyberchondria has rarely been investigated as a factor capable of influencing one’s quality of life (QOL). QOL is defined, by the World Health Organization (WHO), as “an individual's perception of their position in life in the context of the culture and value systems in which they live and in relation to their goals, expectations, standards and concerns” [9]. The COVID-19 pandemic has widely affected human’s life in a relatively short period of time [10]. Lockdown periods forced by governments as safety measures to face this pandemic had negative impact on mental and psychosocial wellbeing, including but not limited to stress, negative emotions and impaired cognition [11]. People are confused how secure they are and how anxious they should be because of the unpredictability of the situation, leading to concerns and misunderstandings [12]. In this sense, the first negative factor that is likely to affect one's quality of life is the fear that arises as a result of COVID-19 [13]. Fear is one of the most basic human emotions, and it is crucial to humanity's evolutionary emotional continuity [14]. Individuals naturally became concerned about the COVID-19 because of the extremely high infection rate and relatively high mortality. Fear of contacting people who may be infected with COVID-19 has been reported, and this, unfortunately, may intensify the damage of the disease itself [15]. People tried to cope in a socially distancing context by engaging in activities that affirm social (e.g., communicating virtually online) in order to overcome perceived threats and contagion fear [16]. These behaviors may serve as coping mechanisms for people who are scared of getting the disease, improving their well-being and therefore their quality of life [16].

According to recent research, during pandemic times, there is a significant risk of seeing secondary effects in any area of society, and emotional and behavioral issues such as anxiety, fear, depression, internet addiction, substance addiction and more [13]. Despite the Internet's usefulness for easily accessing information and encouraging health behaviors, it has been shown that Internet health information searches may expose inexperienced users to possible harms due to self-diagnosis, self-treatment, and growing levels of anxiety about health leading to possible consequent symptoms of depression and distress [17, 18]. Cyberchondria has been strongly associated with increased anxiety about health [19]. Extreme anxiety induces excessive online searching for one's health, and that is more closely linked to health anxiety disorder [20]. In contrast, another theory shows that cyberchondria is related to obsessive compulsive behavior [19]. The compulsive research on health information acquisition contributes to an unhealthy rise in health anxiety [20]. The causality of the association between online searches related to health and health anxiety can differ from person to person. Thus, in order to seek reassurance for their health, users encounter different coping strategies that in some times, cause more discomfort and raise health anxiety even more [20]. Since the syndrome is driven by anxiety regarding potential health conditions or symptoms, the perceived severity of the given situation may be expected to increase [3]. Despite the fact that COVID-19 is positioned as a stressor with broad implications for mental and physical health, individual stress experiences vary greatly. The process of stress and coping maintains this heterogeneity [21]. Person features and contextual realities naturally impact both appraisal and coping behaviors. In general, stressor evaluations are correlated with higher overall levels of stress and its mental health correlates, such as depression and anxiety, as more severe or threatening [21]. A recent study revealed that 1724 US physicians who spent a greater proportion of their workday treating patients with COVID-19 experienced greater symptoms of depression, anxiety, and PTSD [22].

**Fig. 1:**
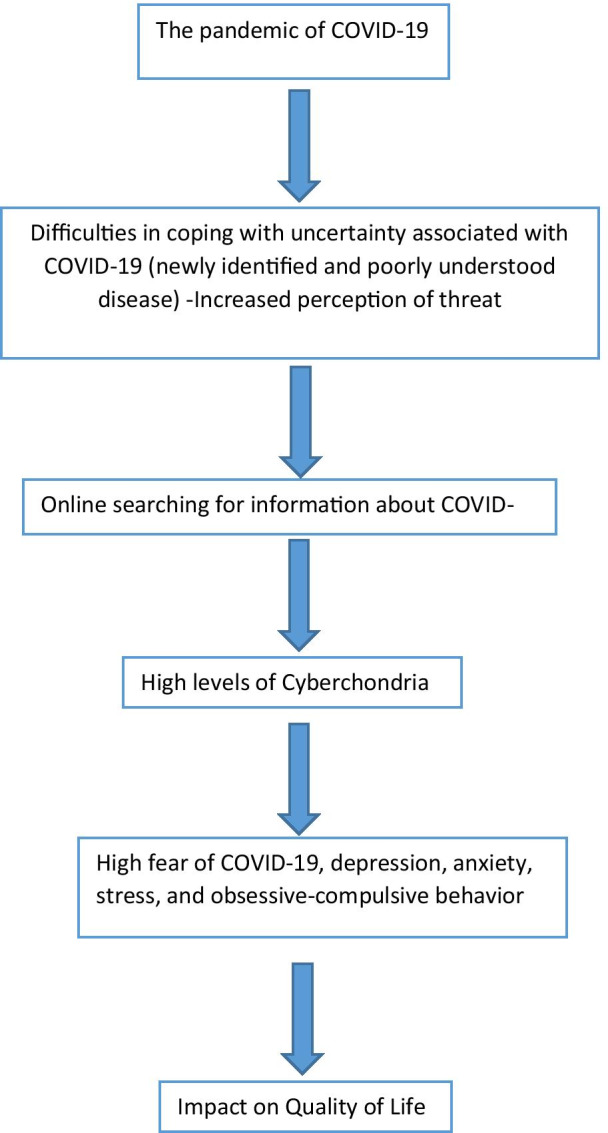
Model of cyberchondria during the COVID-19 pandemic and its impact on the quality of life, along with the mediating effect of fear of COVID-19, depression, anxiety, stress and obsession-compulsion disorders in this association

Many studies have reported the association between the QOL, cyberchondria and the coronavirus pandemic [2, 19, 23]. The proposed association between the pandemic, cyberchondria and the quality of life is illustrated in Fig. 1. No previous studies assessed the mediating effect of fear of COVID-19, depression, anxiety, stress and obsession-compulsion disorders in the association of cyberchondria and QOL. In Lebanon, however, there has been no research taking the quality of life of the population with COVID-19 pandemic along with the presence the cyberchondria. For this purpose, the aim of this study was to assess the association between quality of life of Lebanese community during the COVID-19 and cyberchondria and evaluate the mediating effect of fear of COVID-19, depression, anxiety, stress and obsession-compulsion disorders in this association.

## Methods

### Study design

This cross-sectional study was carried out between December 2020 and January 2021, during the COVID-19 pandemic when lockdown procedures were implemented at different instances, and the measures taken by the government used to change on regular basis according to the severity of COVID-19 situation; 449 persons participated in this study by filling the online questionnaire. The sample was selected using a snowball technique from the five governorates of Lebanon (Beirut, Bekaa, Mount Lebanon, South Lebanon and North Lebanon). Respondents were briefed about the topic, and the different aspects of the questionnaire before filling it out, while being assured of the anonymity of the response. Inclusion criteria included people above 18 years old living in Lebanon. Excluded were those who refused to participate in the study.

### Minimal sample size calculation

The G-power software calculated a minimal sample of 395, based on a 5% alpha error and a power of 80% and 10 factors to be entered in the linear regression models.

### Questionnaire

The questionnaires sent to participants was in the Arabic language, which is the official language of Lebanon as well as the native language of all respondents. The questionnaire included a sociodemographic category, and a scale-based category regarding different factors, as follows:

#### Sociodemographic data

In this part of the questionnaire, participants were asked about their general sociodemographic data, including their age, educational level, marital status, number of children and information about house crowding index of the participants. The household crowding index was calculated by dividing the number of persons living in the house and the number of rooms in the house, excluding the bathroom and the kitchen [24].

#### Scale-based category

The following scales were used in the questionnaire:

##### Cyberchondria severity scale

The 12-item short version scale was developed from the original 33-item Cyberchondria Severity Scale (CSS) [25, 26] to allow for a multidimensional assessment of cyberchondria (compulsion, distress, excessiveness, reassurance, without the mistrust of medical professional part). It is scored on a 5-point Likert type scale (ranging from ‘1 = never’ to ‘5 = always’). The CSS items can be summed to form a total score. Higher scores indicate higher cyberchondria (Cronbach’s alpha in this study = 0.919).

##### Quality of life short form‐12 health survey (SF‐12)

Validated in Lebanon [27], this instrument is composed of twelve items to assess physical and mental health. The scores of each of these two sections are ranged from “0” up to “100”. Greater scores indicate a higher physical/mental quality of life [28]. (Cronbach’s alpha in this study = 0.746 for both subscales).

##### Yale brown obsession compulsion scale (YBOCS)

The Yale-Brown Obsessive Compulsive Scale provides a specific measure of the severity of symptoms of obsessive–compulsive disorder that is not influenced by the type of obsessions or compulsions present. The scale is a 10-item scale, each item rated from 0 (no symptoms) to 4 (extreme symptoms) (total range 0–40), with higher scores indicating more severe obsessive compulsive symptoms [29] (Cronbach’s alpha for the total scale in this study = 0.860). The Arabic version of this scale has been previously used in a previous project [30].

##### Fear of COVID-19 scale (7 items)

The FCV-19S is a unidimensional scale that assesses the fear of COVID-19. The instrument comprises seven items (e.g., “My heart races or palpitates when I think about getting coronavirus-19”) which are responded to on a 5-point Likert scale from 1 (strongly disagree) to 5 (strongly agree). The scores that can be obtained from the FCV-19S vary between 7 and 35, and higher scores indicate greater fear of COVID-19 [15] (Cronbach’s alpha for the total scale in this study = 0.884). The Arabic version of this scale has been previously used in a previous project [31].

##### Lebanese anxiety scale

The LAS-10 is a 10-item scale used to screen for anxiety in the general Lebanese adult [32] and adolescent [33] populations. Questions 1–7 are scored on a 5-point Likert scale from 0 (not present) to 4 (very severe), while items 8–10 are graded on a 4-point Likert scale from 1 (never/almost never) to 4 (almost always) [32]. Higher scores indicate higher anxiety (Cronbach’s alpha in this study = 0.928).

##### Beirut distress scale

The BDS-10 [34] evaluates the level of stress in the general Lebanese adult population. It consists of 22 questions exploring six different domains: depressive symptoms, demotivation, psychosomatic symptoms, mood deterioration, intellectual inhibition, and anxiety. Responses are rated on a 4-point Likert scale from 0 (not at all) to 3 (all of the times), with higher scores indicating higher levels of stress [35] (Cronbach’s alpha for the total scale in this study = 0.892).

##### Montgomery and Asberg Depression Rating Scale

The Montgomery–Åsberg Depression Rating Scale (MADRS) [36], validated in Lebanon [37], is a ten-item diagnostic questionnaire which psychiatrists use to measure the severity of depressive episodes in patients with mood disorders. MADRS items are rated on a 0–6 continuum (0 = no abnormality, 6 = severe). Higher scores indicate higher depressive manifestations. (Cronbach’s alpha for the total scale in this study = 0.889).

### Translation procedure

A clinical psychologist performed the forward translation from English into Arabic for the cyberchondria scale. A professional medical writer verified this translation. The backward translation was performed by a second clinical psychologist, unaware of the scales’ notions and fluent in Arabic [38]. The principal investigator matched the back-translated English questionnaire with the original one to detect inconsistencies and solve discrepancies between the two versions [38].

### Statistical analysis

The SPSS software version 25 was used for the statistical analysis. Three factor analyses were initiated to confirm the legitimacy of the construct of the cyberchondria severity, fear of COVID-19 and YBOCS scales in our sample. The Kaiser–Meyer–Olkin (KMO) value and the Bartlett's sphericity test were checked for sampling adequacy. The factors with Eigen values > 1 were kept. Reliability was checked using Cronbach's alpha for the total scales. The sample was normally distributed as verified by the skewness and kurtosis of the PCS QOL and MCS QOL scores, which varied between − 2 and + 2 [39]. These conditions consolidate the assumptions of normality in samples larger than 300 [40]. The Student t and ANOVA tests were used to compare two and three or more means respectively. Pearson correlation test was used to correlate two continuous variables. Linear regressions were conducted, taking the physical and mental QOL scores as dependent variables. Independent variables entered in the multivariable analyses and mediation models were those that showed a *p* < 0.2 in the bivariate analysis. Significance was set at a *p* < 0.05.

The SPSS AMOS software v.24 was used to conduct the structural equation modeling of the association between cyberchondria severity and physical/mental quality of life, taking different mediators into account (anxiety, stress, depression, obsessive–compulsive disorder and fear of COVID-19). Multiple indices of goodness-of-fit were described: the Relative Chi-square (χ^2^/*df*) (cut-off values: < 2–5), the Root Mean Square Error of Approximation (RMSEA) (close and acceptable fit are considered for values < 0.05 and < 0.11 respectively), the Tucker Lewis Index (TLI) and the Comparative Fit Index (CFI) (acceptable values are ≥ 0.90) [41, 42].

## Results

### Sociodemographic and other characteristics of the participants

A total of 449 participants out of 590 (76.10%) approached agreed to enroll in this study. The mean age of the sample was 24.34 ± 8.22 years, with 70.6% females. The mean cyberchondria severity score was 15.91 ± 9.64, whereas the mean physical and mental QOL scores were 38.98 ± 7.44 and 33.88 ± 8.45 respectively. Table 1 includes more details about the sample.

**Table 1:** Sociodemographic and other characteristics of the participants (N = 449)

Variable	N (%)
Gender	
Male	132 (29.4%)
Female	317 (70.6%)
Marital status	
Single/widowed/divorced	364 (81.1%)
Married	85 (18.9%)
Education level	
Complementary or less	34 (7.6%)
Secondary	61 (13.6%)
University	354 (78.8%)

	Mean ± SD
Age (in years)	24.34 ± 8.22
Number of children	0.61 ± 1.36
Cyberchondria severity score	15.91 ± 9.64
Physical QOL score	38.98 ± 7.44
Mental QOL score	33.88 ± 8.45
Anxiety	20.29 ± 9.59
Depression	15.50 ± 13.13
Stress	11.25 ± 6.98
Obsession compulsive disorder (YBOCS score)	10.19 ± 8.38
Fear of COVID-19 score	15.64 ± 6.14

### Factor analyses

The results of the factor analyses of the cyberchondria severity scale (Table 2, Model 1), YBOCS scale (Table 2, Model 2) and Fear of COVID-19 scale (Table 2, Model 3) showed that all scales’ items were extracted. The factor loadings, KMO values, the p-values of the Bartlett’s test of sphericity and communalities are summarized in Table 2.

**Table 2:** Factor analysis of the different scales

Variable	Factor 1			Communality
*Model 1: Cyberchondria severity scale*				
1. If I notice an unexplained bodily sensation I will search for it on the internet	0.591			0.349
2. Researching symptoms or perceived medical conditions online distract me from reading news/sports/entertainment articles online	0.670			0.449
3. I read different web pages about the same perceived condition	0.725			0.525
4. I start to panic when I read online that a symptom I have is found in a rare/serious condition	0.728			0.530
5. Researching symptoms or perceived medical conditions online lead me to consult with my GP	0.683			0.467
6. I enter the same symptoms into a web search on more than one occasion	0.764			0.584
7. Researching symptoms or perceived medical conditions online interrupt my work (e.g. writing emails, working on word documents or spreadsheets)	0.822			0.675
8. I think I am fine until I read about a serious condition online	0.745			0.555
9. I feel more anxious or distressed after researching symptoms or perceived medical conditions online	0.748			0.560
10. Researching symptoms or perceived medical conditions online interrupt my offline social activities (e.g., reduces time spent with friends/family)	0.782			0.611
11. I suggest to my GP/medical professional that I may need a diagnostic procedure that I read about online (e.g., a biopsy/ a specific blood test)	0.744			0.553
12. Researching symptoms or perceived medical conditions online lead me to consult with other medical specialists (e.g., consultants)	0.742			0.550
KMO = 0.923, Bartlett’s test of sphericity *p* < 0.001; Percentage of variance explained = 53.41%				

Variable	Factor 1	Factor 2	Factor 3	Communality
*Model 2: YBOCS scale (using a promax rotation)*				
1. Time occupied by obsessive thoughts		0.770		
2. Interference due to obsessive thoughts		0.800		
3. Distress associated with obsessive thoughts		0.922		
4. Resistance against obsessions			0.900	
5. Degree of control over obsessive thoughts		0.483		
6. Time spent performing compulsive behaviors	0.839			
7. Interference due to compulsive behaviors	0.922			
8. Distress associated with compulsive behavior	0.672			
9. Resistance against compulsions			0.811	
10. Degree of control over compulsive behavior	0.726			
Percentage of variance explained	46.07	11.31	10.07	
KMO = 0.841, Bartlett’s test of sphericity *p* < 0.001; Percentage of total variance explained = 67.45%				

Variable	Factor 1	Factor 2		Communality
*Model 3: Fear of COVID-19 scale (using a promax rotation)*				
1. I am most afraid of Corona		1.018		0.810
2. It makes me uncomfortable to think about Corona		0.852		0.774
3. My hands become clammy when I think about Corona	0.793			0.744
4. I am afraid of losing my life because of Corona		0.561		0.616
5. When I watch news and stories about Corona on social media, I become nervous or anxious		0.633		0.699
6. I cannot sleep because I’m worrying about getting Corona	0.953			0.782
7. My heart races when I think about getting Corona	0.895			0.782
Percentage of variance explained	59.43	14.97		
KMO = 0.838, Bartlett’s test of sphericity *p* < 0.001; Percentage of total variance explained = 74.40%				

### Bivariate analysis

Higher means physical and mental QOL scores were significantly found in those with a university level of education compared to the other levels (Table 3). Higher anxiety, depression, stress, cyberchondria severity, obsession-compulsion and fear of COVID-19 were significantly associated with lower physical QOL scores. Moreover, higher anxiety, depression, cyberchondria severity and fear of COVID-19 were significantly associated with lower mental QOL scores (Table 4).

**Table 3:** Bivariate analysis of categorical factors associated with the physical and mental QOL scores

Variable	Physical QOL score	Mental QOL score
Gender		
Male	38.95 ± 7.27	33.88 ± 9.11
Female	38.99 ± 7.52	33.88 ± 8.18
*P*	0.959	0.996
Effect size	0.005	0.001
Marital status		
Single/widowed/divorced	39.04 ± 7.47	33.73 ± 8.58
Married	38.70 ± 7.36	34.51 ± 7.94
*P*	0.703	0.449
Effect size	0.046	0.093
Education level		
Complementary or less	37.63 ± 6.20	31.65 ± 7.41
Secondary	37.15 ± 7.27	32.14 ± 9.51
University	39.42 ± 7.54	34.40 ± 8.330
*P*	**0.048**	**0.043**
Effect size	0.117	0.119

Numbers in bold indicate significant *p*-values

**Table 4:** Bivariate analysis of continuous variables associated with the physical and mental QOL scores

Variable	Physical QOL score	Mental QOL score
Age (in years)	0.103^c^	0.036
Number of children	− 0.014	− 0.003
Household crowding index	− 0.042	− 0.028
Anxiety	− 0.149^b^	− 0.427^a^
Depression	− 0.202^a^	− 0.194^a^
Stress	− 0.239^a^	0.002
Cyberchondria severity score	− 0.132^b^	− 0.101^c^
Obsession Compulsive Disorder (YBOCS score)	− 0.219^a^	− 0.064
Fear of COVID-19 score	− 0.167^a^	− 0.113^c^

^a^*p* < 0.001; ^b^*p* < 0.01; ^c^*p* < 0.05

### Multivariable analysis

Having a university level of education compared to complementary or less (B = 2.53), and older age (B = 0.09) were significantly associated with higher physical QOL scores, whereas higher obsession-compulsion disorder (higher YBOCS scores) (B =  − 0.23), higher stress (B =  − 0.24) and higher anxiety (B =  − 0.16) were significantly associated with lower physical QOL scores (Table 5, Model 1).

**Table 5:** Multivariable analysis taking the physical and mental QOL scores as dependent variables

Variable	UB	SB	*p*	95% CI
*Model 1: Physical QOL score as the dependent variable*				
Stress	− 0.24	− 0.23	< 0.001	− 0.34 to − 0.15
Obsession-compulsion disorder	− 0.23	− 0.25	< 0.001	− 0.31 to − 0.15
Anxiety	− 0.16	− 0.21	< 0.001	− 0.23 to − 0.09
University level of education vs complementary or less*	2.53	0.14	0.002	0.91–4.14
Age	0.09	0.10	0.037	0.01–0.17
Variables entered in the model: education, Age, anxiety, depression, stress, cyberchondria severity, obsession-compulsion disorder, fear of COVID				
*Model 2: Mental QOL score as the dependent variable*				
Anxiety	− 0.38	− 0.43	< 0.001	− 0.45 to − 0.30
Variables entered in the model: education, anxiety, depression, cyberchondria severity, fear of COVID				

Numbers in bold indicate significant *p*-values

Nagelkerke R^2^ = 16.7% for model 1 and 18.2% for model 2; QOL = Quality of life

Higher anxiety (B =  − 0.38) was significantly associated with lower mental QOL scores (Table 5, Model 2).

### Structural equation modeling

**Fig. 2:**
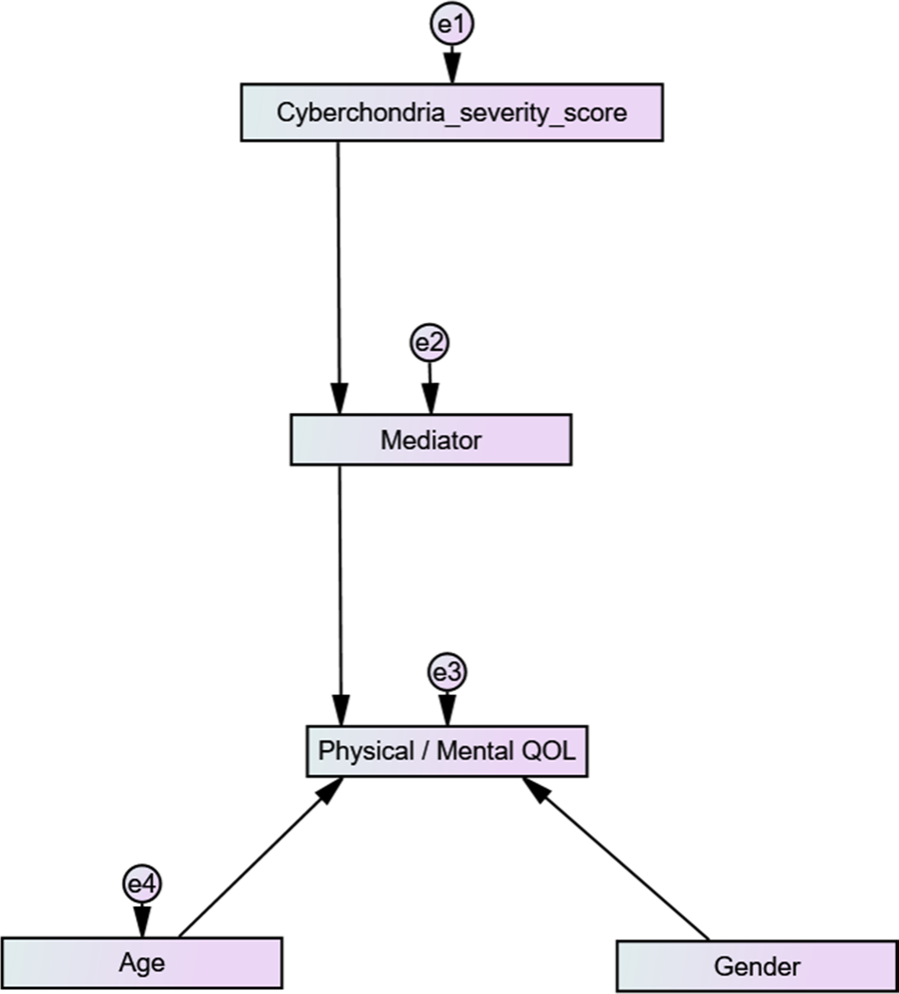
Structural equation model of the association between cyberchondria severity and physical/mental quality of life, taking different mediators into account (anxiety, stress, depression, obsessive–compulsive disorder and fear of COVID-19).  —observed variable;  —latent variable;  —impact of one variable on another; e—residual error in the prediction of an unobserved factor

The model tested during the structural equation modeling is summarized in Fig. 2.

The fit indices of each SEM are summarized in Table 6, whereas the coefficients with standard errors and *p*-values of the direct effects of variables on each other are summarized in Table 7 (physical QOL) and Table 8 (mental QOL) respectively. Stress, fear of COVID-19 and to a lesser limit OCD, mediated the association between cyberchondria severity and physical QOL, whereas anxiety, stress and fear of COVID-19 mediated the association between cyberchondria severity and mental QOL.

**Table 6:** Fit indices of the structural equation modeling of the association between cyberchondria severity and physical/mental quality of life

Mediator	χ^2^/*df*	TLI	CFI	RMSEA	90% CI	
*Model 1: Physical quality of life taken as the dependent variable*						
Anxiety	17.60/6 = 2.93	0.347	0.608	0.066	0.310	0.102
Stress	6.18/6 = 1.03	0.997	0.998	0.008	0.001	0.062
Depression	11.59/6 = 1.93	0.640	0.784	0.046	0.001	0.085
OCD	9.91/6 = 1.65	0.800	0.880	0.038	0.001	0.079
Fear of COVID-19	11.67/6 = 1.95	0.860	0.920	0.046	0.001	0.085
*Model 2: Mental quality of life taken as the dependent variable*						
Anxiety	13.29/6 = 2.22	0.880	0.930	0.050	0.011	0.090
Stress	10.18/6 = 1.70	0.890	0.930	0.040	0.001	0.080
Depression	10.75/6 = 1.79	0.600	0.760	0.042	0.001	0.082
OCD	10.19/6 = 1.70	0.180	0.510	0.039	0.001	0.080
Fear of COVID-19	10.87/6 = 1.81	0.860	0.920	0.043	0.001	0.082

**Table 7:** Coefficient, standard error and *p*-value of the structured equation modeling (SEM) approach model taking the physical quality of life (PCS) as the dependent variable

Variable	Coefficient	Standard error	*p*
*Model 1: Anxiety as the mediator*			
Cyberchondria → Anxiety	− 0.14	0.05	**0.003**
Anxiety → PCS	− 0.14	0.04	**0.003**
Age → PCS	0.09	0.04	0.07
Gender → PCS	0.01	0.76	0.864
*Model 2: Stress as the mediator*			
Cyberchondria → Stress	0.35	0.03	**< 0.001**
Stress → PCS	0.24	0.05	**< 0.001**
Age → PCS	0.10	0.04	**0.031**
Gender → PCS	− 0.003	0.75	0.947
*Model 3: Depression as the mediator*			
Cyberchondria → Depression	0.05	0.06	0.310
Depression → PCS	0.20	0.03	**< 0.001**
Age → PCS	0.10	0.04	**0.033**
Gender → PCS	0.01	0.75	0.817
*Model 4: OCD as the mediator*			
Cyberchondria → OCD	0.10	0.04	**0.015**
OCD → PCS	0.22	0.04	**< 0.001**
Age → PCS	0.10	0.04	**0.034**
Gender → PCS	0.004	0.75	0.925
*Model 5: Fear of COVID-19 as the mediator*			
Cyberchondria → Fear of COVID-19	0.33	0.03	**< 0.001**
Fear of COVID-19 → PCS	0.16	0.06	**< 0.001**
Age → PCS	0.09	0.04	0.061
Gender → PCS	− 0.004	0.76	0.938

Numbers in bold indicate significant *p*-values

**Table 8:** Coefficient, standard error and *p*-value of the structured equation modeling (SEM) approach model taking the mental quality of life (MCS) as the dependent variable

Variable	Coefficient	Standard error	*p*
*Model 1: Anxiety as the mediator*			
Cyberchondria → Anxiety	− 0.14	0.05	**0.003**
Anxiety → MCS	− 0.43	0.04	**< 0.001**
Age → MCS	− 0.02	0.04	0.606
Gender → MCS	0.01	0.79	0.903
*Model 2: Stress as the mediator*			
Cyberchondria → Stress	0.35	0.03	**< 0.001**
Stress → MCS	0.001	0.06	0.980
Age → MCS	0.04	0.05	0.442
Gender → MCS	0.002	0.88	0.970
*Model 3: Depression as the mediator*			
Cyberchondria → Depression	0.05	0.06	0.310
Depression → MCS	− 0.20	0.03	**< 0.001**
Age → MCS	0.04	0.05	0.376
Gender → MCS	− 0.002	0.86	0.969
*Model 4: OCD as the mediator*			
Cyberchondria → OCD	0.11	0.04	**0.015**
OCD → MCS	− 0.07	0.05	0.167
Age → MCS	0.04	0.05	0.419
Gender → MCS	0.003	0.87	0.956
*Model 5: Fear of COVID-19 as the mediator*			
Cyberchondria → Fear of COVID-19	0.33	0.03	** < 0.001**
Fear of COVID-19 → MCS	0.11	0.07	**0.019**
Age → MCS	0.03	0.05	0.593
Gender → MCS	− 0.01	0.87	0.907

Numbers in bold indicate significant *p*-values

## Discussion

This study demonstrated that higher obsession-compulsion disorder, higher stress and higher anxiety were significantly associated with lower physical QOL scores. Higher anxiety was significantly associated with lower mental QOL scores.

In this study, we found that high level of education was associated with high physical quality of life, corroborating the findings of a previous study [43]. Individuals who have completed higher education expand their chances to secure a career. In general, higher levels of educational attainment are related to better job opportunities and higher income, thereby having a positive impact on the quality of life of an individual [44].

In addition, older age was significantly associated with high levels of physical QOL. Results from previous research about this association, are consistent; while a previous study showed that younger age had a negative impact on quality of life [45], another research showed that higher physical QOL were reported in the group aged 79–90 [46]. A possible reason is that age offers opportunities to develop resilience due to exposure over time to various and distinct stressors, resulting in improved emotional regulation and decreased symptoms of anxiety and depression [45].

We found that obsession-compulsion disorder, stress, and anxiety were significantly associated with lower physical QOL. A previous study found that all aspects of QOL are substantially impaired in individuals with Obsession Compulsion Disorder (OCD) and associated with the severity of the disorder [47]. Given the significant impact of OCD on patients' quality of life and the high frequencies of mental comorbidities, the current COVID-19 epidemic poses a special threat to OCD patients because of the increased disability caused by an increase in the frequency of obsessions and compulsions [47]. Furthermore, a previous study reported that stressful life events have an adverse effect on physical QOL [48]. An earlier study found that anxiety and stress were among the most common symptoms seen during the COVID pandemic [49, 50]. Increased rates of stress and anxiety can be caused by the existence of uncertainty about the disease, reduced contact between individuals, and temporary restrictions of rights and freedoms due to lockdowns [51].

For the mental QOL, we found that high anxiety was associated with low mental QOL. During the COVID-19 epidemic, a recent study looked at the prevalence and determinants of anxiety symptoms, found that 41% of the sample reported mild symptoms of anxiety whereas 23% revealed severe anxiety symptoms [45]. Key aspects of anxiety included financial issues, personal health concerns, satisfaction with the current state of QOL and current health levels [45]. Another study found that the population is at risk of experiencing anxiety because of the high rate of mortality, insecurity of resources and food, experience of infected people, which may lead to certain adverse mental health issues [52]. In fact, the pandemic has likely affected mental health in a number of ways, particularly with widespread social isolation resulting from required safety measures. Social isolation and loneliness led to both poor mental and physical health and high levels of anxiety [53].

We found that stress, fear of COVID-19 and to a lesser limit OCD, mediated the association between cyberchondria severity and physical QOL, whereas anxiety, stress and fear of COVID-19 mediated the association between cyberchondria severity and mental QOL. Well-being and life satisfaction have been adversely impacted by the new pandemic of COVID-19 [16]. The findings of a latest study found that there was a marked rise in pandemic issues and major behavioral changes [54]. Those behavioral changes increase the sense of restricted freedom, psychological distress and community anxiety. Therefore, the situation's unpredictability leaves people unsure about how secure they are and how concerned they should be, raising fears of the virus [54]. Studies have also shown that since the COVID-19 pandemic, the use of the internet and social networking sites for stress reduction, has increased [54]. Another symptom encountered is that prolonged internet search triggers COVID-19 fears at high levels, leading to severe health problems [54]. Indeed, a large part of the literature considers the role of disease event-related fears as predictors or mediators of QOL indicators [16]. People, like in every other pandemic situation, are first and foremost afraid of their lives, followed by the fear of losing their belongings. As the fear grows stronger, depression develops, and both fear and sadness are linked to different types of anxiety [55]. Although fears can adversely impact the QOL, it can also encourage people to reduce health-threatening behaviors [16]. In addition, the exacerbation of obsessive–compulsive behavior has been well-documented during past outbreaks. Nonetheless, the current COVID-19 epidemic has shown that obsessive–compulsive behavior has a significant effect on quality of life especially for OCD patients who already have concerns about sanitary measures and obsessive cleaning [47].

Moreover, a finding consistent with ours is that cyberchondria's impact during COVID-19 was significantly mediated by anxiety [54]. 28% of the participants in an Indian study were moderate to extremely severely anxious [56]. People with elevated anxiety may assume that their feelings and symptoms are harmful. Following what they have read on the internet, they begin over-searching and worrying about their health. As a result, by looking online for appropriate medical information to minimize their harmful feelings, they try to find the source of these feelings [54]. For this purpose, exposure to social media and prolonged internet searching was associated with significant increase in anxiety symptoms [2].

This study highlights the significant association between cyberchondria and quality of life among the Lebanese population in the time of COVID-19. To our knowledge, no previous research has looked into a direct link between cyberchondria and quality of life; however, it could be mediated by other factors including depression, anxiety, or mental health issues as found in this study. Further studies are needed in order to better understanding the mechanism between cyberchondria and QOL. Thus, by shedding the lights on the findings of the research, we can assume that social support is crucial in reducing the negative effects of stress, anxiety, and fear, as well as promoting adaptation and, as a result, enhancing QOL during these hard times. A follow-up study evaluating how this population is reacting to the future trajectory of the COVID-19 pandemic, assessing the long-term impacts of the pandemic and investigating resources and resilience factors is much needed in order to cope better.

This study has some limitations. The data's cross-sectional nature limits the ability to pull causal conclusions. The use of a self-administered questionnaire and the under or over-estimation of a question by a participant, poses a risk for information bias. There is also a risk of selection bias, given the nature of the sample based on which the study was conducted. Since the sample is small, further studies with larger sample are required in order to better assess the associations in this study. Some scales are not validated in Lebanon, thus, results should be interpreted with caution. Residual confounding bias is also possible since not all factors associated with QOL were considered in this study.

## Conclusion

This research reported interesting results encouraging more exploration of cyberchondria and its association with quality of life during this unique period of the pandemic. This study adds useful information to the literature and shows similar results to recent studies found worldwide. However, this virus has altered the lives of individuals all across the world, and its consequences will last for a long time. Along with all of the steps done to stop the development of COVID-19 and improve physical outcomes, mental health requires immediate care. More research is needed to determine the coping techniques people are employing to deal with the pandemic. In addition, qualitative research could lead to a better understanding of the coronavirus's impact on people's physical and mental health.

## Data Availability

There is no public access to all data generated or analyzed during this study to preserve the privacy of the identities of the individuals. The dataset that supports the conclusions is available to the corresponding author upon request.

## Abbreviations

BDS-10: Beirut Distress Scale
CI: Confidence intervals
COVID-19: Corona virus disease 2019
CSS: Cyberchondria Severity Scale
LAS-10: Lebanese Anxiety Scale
MADRS: Montgomery–Åsberg Depression Rating Scale
OCD: Obsessive compulsion disorder
OHR: Online health research
QOL: Quality of life
SF‐12: Quality of Life Short Form‐12 Health Survey
WHO: World Health Organization
YBOCS: Yale Brown Obsession Compulsion Scale

## References

[1] Jungmann SM, Witthöft M (2020). Health anxiety, cyberchondria, and coping in the current COVID-19 pandemic: Which factors are related to coronavirus anxiety?. J Anxiety Disord.

[2] Jokic-Begic N, Lauri Korajlija A, Mikac U (2020). Cyberchondria in the age of COVID-19. PLOS ONE.

[3] Farooq A, Laato S, Islam AN (2020). Impact of online information on self-isolation intention during the COVID-19 pandemic: cross-sectional study. J Med Internet Res.

[4] Zheng H, Tandoc Jr EC: Calling Dr. Internet: analyzing news coverage of cyberchondria. Journal Pract. 2020; 1–17

[5] Osei Asibey B, Agyemang S, Boakye Dankwah A (2017). The internet use for health information seeking among Ghanaian university students: a cross-sectional study. Int J Telemed Appl.

[6] Laato S, Islam AN, Islam MN, Whelan E (2020). What drives unverified information sharing and cyberchondria during the COVID-19 pandemic?. Eur J Inf Syst.

[7] Mathes BM, Norr AM, Allan NP, Albanese BJ, Schmidt NB (2018). Cyberchondria: Overlap with health anxiety and unique relations with impairment, quality of life, and service utilization. Psychiatry Res.

[8] Starcevic V, Berle D, Arnáez S (2020). Recent insights into cyberchondria. Curr Psychiatry Rep.

[9] Measuring Quality of Life https://www.who.int/tools/whoqol#:~:text=WHO%20defines%20Quality%20of%20Life,%2C%20expectations%2C%20standards%20and%20concerns

[10] Guo D, Han B, Lu Y, Lv C, Fang X, Zhang Z, Liu Z, Wang X (2020). Influence of the COVID-19 pandemic on quality of life of patients with Parkinson’s Disease. Parkinson’s Disease.

[11] Slimani M, Paravlic A, Mbarek F, Bragazzi NL, Tod D (2020). The relationship between physical activity and quality of life during the confinement induced by COVID-19 outbreak: a pilot study in Tunisia. Front Psychol.

[12] Alyami M, De Albuquerque JV, Krägeloh CU, Alyami H, Henning MA: Effects of fear of COVID-19 on mental well-being and quality of life: a path analysis. 2020. 10.4103/sjmms.sjmms_630_20 PMC7839565 33519340

[13] Seçer İ, Ulaş S, Karaman-Özlü Z (2020). The Effect of the Fear of COVID-19 on Healthcare Professionals’ Psychological Adjustment Skills: Mediating Role of Experiential Avoidance and Psychological Resilience. Frontiers in Psychology.

[14] Yalçın İ, Can N, Mançe Çalışır Ö, Yalçın S, Çolak B: Latent profile analysis of COVID-19 fear, depression, anxiety, stress, mindfulness, and resilience. Curr Psychol (New Brunswick, NJ) 2021; 1–11. 10.1007/s12144-021-01667-x PMC8012016 33821112

[15] Ahorsu DK, Lin C-Y, Imani V, Saffari M, Griffiths MD, Pakpour AH: The fear of COVID-19 scale: development and initial validation. Int J Mental Health Addict. 2020; 1–9. 10.1007/s11469-020-00270-8 PMC7100496 32226353

[16] Lardone A, Sorrentino P, Giancamilli F, Palombi T, Simper T, Mandolesi L, Lucidi F, Chirico A, Galli F (2020). Psychosocial variables and quality of life during the COVID-19 lockdown: a correlational study on a convenience sample of young Italians. PeerJ.

[17] Marino C, Fergus TA, Vieno A, Bottesi G, Ghisi M, Spada MM (2020). Testing the Italian version of the Cyberchondria Severity Scale and a metacognitive model of cyberchondria. Clin Psychol Psychother.

[18] Gioia F, Boursier V (2020). What does predict cyberchondria? evidence from a sample of women. J Psychol.

[19] Starcevic V, Schimmenti A, Billieux J, Berle D: Cyberchondria in the time of the COVID‐19 pandemic. Hum Behav Emerg Technol 2020. 10.1002/hbe2.233 PMC7753572 33363277

[20] Erdoğan A, Hocaoğlu Ç (2020). Cyberchondria: a review. Psikiyatride Guncel Yaklasimlar.

[21] Whitehead BR (2021). COVID-19 as a stressor: pandemic expectations, perceived stress, and negative affect in older adults. J Gerontol Ser B.

[22] Gainer DM, Nahhas RW, Bhatt NV, Merrill A, McCormack J (2021). Association between proportion of workday treating COVID-19 and depression, anxiety, and PTSD outcomes in US physicians. J Occup Environ Med.

[23] Adıbelli D, Sümen A (2020). The effect of the coronavirus (COVID-19) pandemic on health-related quality of life in children. Children and Youth Services Review.

[24] Melki I, Beydoun H, Khogali M, Tamim H, Yunis K (2004). Household crowding index: a correlate of socioeconomic status and inter-pregnancy spacing in an urban setting. J Epidemiol Commun Health.

[25] Fergus TA (2014). The Cyberchondria Severity Scale (CSS): an examination of structure and relations with health anxiety in a community sample. J Anxiety Disord.

[26] McElroy E, Shevlin M (2014). The development and initial validation of the cyberchondria severity scale (CSS). J Anxiety Disord.

[27] Haddad C, Sacre H, Obeid S, Salameh P, Hallit S (2021). Validation of the Arabic version of the "12-item short-form health survey" (SF-12) in a sample of Lebanese adults. Arch Public Health.

[28] Ware J, Kosinski M, Keller S: SF-36 physical and mental health summary scales. A User's Manual 2001; 1994.

[29] Goodman WK, Price LH, Rasmussen SA, Mazure C, Fleischmann RL, Hill CL, Heninger GR, Charney DS (1989). The Yale–Brown obsessive compulsive scale. I. Development, use, and reliability. Arch Gen Psychiat.

[30] Wehbe J, Haddad C, Obeid S, Hallit S, Haddad G (2019). Prevalence of obsessive-compulsive disorder in patients with schizophrenia and outcome on positive and negative symptoms, cognition, and quality of life. J Nerv Ment Dis.

[31] Bitar Z, Haddad C, Obeid S, Hallit S (2021). Treatment satisfaction and its association with anxiety, depression and fear of COVID-19 among Lebanese inpatients with schizophrenia. Pharm Pract (Granada).

[32] Hallit S, Obeid S, Haddad C, Hallit R, Akel M, Haddad G, Soufia M, Khansa W, Khoury R, Kheir N (2020). Construction of the Lebanese anxiety scale (LAS-10): a new scale to assess anxiety in adult patients. Int J Psychiatry Clin Pract.

[33] Merhy G, Azzi V, Salameh P, Obeid S, Hallit S (2021). Anxiety among Lebanese adolescents: scale validation and correlates. BMC Pediatr.

[34] Malaeb D, Farchakh Y, Haddad C, Sacre H, Obeid S, Hallit S, Salameh P: Validation of the Beirut Distress Scale (BDS-10), a short version of BDS-22, to assess psychological distress among the Lebanese population. Perspect Psych Care. 2021. 10.1111/ppc.12787 33821486

[35] Salameh P, Aline H, Badro DA, Abou Selwan C, Randa A, Sacre H (2020). Mental health outcomes of the COVID-19 pandemic and a collapsing economy: perspectives from a developing country. Psychiatry Research.

[36] Åsberg ME, Perris CE, Schalling DE, Sedvall GE: CPRS: Development and applications of a psychiatric rating scale. Acta Psychiatrica Scandinavica. 1978. 10.1111/j.1600-0447.1978.tb02357.x 277059

[37] Hallit S, Obeid S, El Hage W, Kazour F (2019). Validation of the Arabic version of the MADRS scale among Lebanese patients with depression. L'Encephale.

[38] Kasrine Al Halabi C, Obeid S, Sacre H, Akel M, Hallit R, Salameh P, Hallit S (2021). Attitudes of Lebanese adults regarding COVID-19 vaccination. BMC Public Health.

[39] George D: SPSS for windows step by step: a simple study guide and reference, 17.0 update, 10/e: Pearson Education India; 2011.

[40] Mishra P, Pandey CM, Singh U, Gupta A, Sahu C, Keshri A (2019). Descriptive statistics and normality tests for statistical data. Ann Card Anaesth.

[41] Byrne BM (2013). Structural equation modeling with Mplus: basic concepts, applications, and programming.

[42] Marsh HW, Hau K-T, Wen Z (2004). In search of golden rules: comment on hypothesis-testing approaches to setting cutoff values for fit indexes and dangers in overgeneralizing Hu and Bentler's (1999) findings. Struct Equ Model.

[43] Gil-Lacruz M, Gil-Lacruz AI, Gracia-Pérez ML (2020). Health-related quality of life in young people: the importance of education. Health Qual Life Outcomes.

[44] Education in the context of quality of life https://ec.europa.eu/eurostat/statistics-explained/index.php/Quality_of_life_indicators_-_education#Education_in_the_context_of_quality_of_life

[45] Solomou I, Constantinidou F (2020). Prevalence and predictors of anxiety and depression symptoms during the COVID-19 pandemic and compliance with precautionary measures: age and sex matter. Int J Environ Res Public Health.

[46] Brett CE, Dykiert D, Starr JM, Deary IJ (2019). Predicting change in quality of life from age 79 to 90 in the Lothian Birth Cohort 1921. Qual Life Res.

[47] Benatti B, Albert U, Maina G, Fiorillo A, Celebre L, Girone N, Fineberg N, Bramante S, Rigardetto S, Dell’Osso B (2020). What happened to patients with obsessive compulsive disorder during the COVID-19 pandemic? a multicentre report from tertiary clinics in northern Italy. Front Psych.

[48] Zhang H, Zhang Q, Gao T, Kong Y, Qin Z, Hu Y, Cao R, Mei S (2019). Relations between stress and quality of life among women in late pregnancy: the parallel mediating role of depressive symptoms and sleep quality. Psychiatry Investig.

[49] Özdin S, Bayrak Özdin Ş (2020). Levels and predictors of anxiety, depression and health anxiety during COVID-19 pandemic in Turkish society: the importance of gender. Int J Soc Psychiatry.

[50] El Othman R, Touma E, El Othman R, Haddad C, Hallit R, Obeid S, Salameh P, Hallit S (2021). COVID-19 pandemic and mental health in Lebanon: a cross-sectional study. Int J Psychiatry Clin Pract.

[51] Ozdemir F, Cansel N, Kizilay F, Guldogan E, Ucuz I, Sinanoglu B, Colak C, Cumurcu HB (2020). The role of physical activity on mental health and quality of life during COVID-19 outbreak: a cross-sectional study. Eur J Integr Med.

[52] Zhang Y, Ma ZF (2020). Impact of the COVID-19 pandemic on mental health and quality of life among local residents in Liaoning Province, China: a cross-sectional study. Int J Environ Res Public Health.

[53] Panchal N, Kamal R, Orgera K, Cox C, Garfield R, Hamel L, Chidambaram P (2020). The implications of COVID-19 for mental health and substance use.

[54] Hashemi SGS, Hosseinnezhad S, Dini S, Griffiths MD, Lin C-Y, Pakpour AH (2020). The mediating effect of the cyberchondria and anxiety sensitivity in the association between problematic internet use, metacognition beliefs, and fear of COVID-19 among Iranian online population. Heliyon.

[55] Mahmud MS, Talukder MU, Rahman SM (2020). Does ‘Fear of COVID-19’ trigger future career anxiety? an empirical investigation considering depression from COVID-19 as a mediator. Int J Soc Psychiatry.

[56] Verma S, Mishra A (2020). Depression, anxiety, and stress and socio-demographic correlates among general Indian public during COVID-19. Int J Soc Psychiatry.

